# ASTEROID stereotest v1.0: lower stereo thresholds using smaller, denser and faster dots

**DOI:** 10.1111/opo.12737

**Published:** 2020-09-29

**Authors:** Jenny C A Read, Zhen Yi Wong, Xinye Yek, Ying Xin Wong, Omar Bachtoula, Ichasus Llamas-Cornejo, Ignacio Serrano-Pedraza

**Affiliations:** 1 Biosciences Institute, Newcastle University, Newcastle upon Tyne, UK; 2 Faculty of Psychology, Universidad Complutense de Madrid, Madrid, Spain

**Keywords:** binocular vision, psychophysics, stereoacuity, stereopsis, stereoscopic vision, stereotest, vision tests

## Abstract

**Purpose:**

In 2019, we described ASTEROID, a new stereotest run on a 3D tablet computer which involves a four-alternative disparity detection task on a dynamic random-dot stereogram. Stereo thresholds measured with ASTEROID were well correlated with, but systematically higher than (by a factor of around 1.5), thresholds measured with previous laboratory stereotests or the Randot Preschool clinical stereotest. We speculated that this might be due to the relatively large, sparse dots used in ASTEROID v0.9. Here, we introduce and test the stereo thresholds and test-repeatability of the new ASTEROID v1.0, which uses precomputed images to allow stereograms made up of much smaller, denser dots.

**Methods:**

Stereo thresholds and test/retest repeatability were tested and compared between the old and new versions of ASTEROID (*n* = 75) and the Randot Circles (*n* = 31) stereotest, in healthy young adults.

**Results:**

Thresholds on ASTEROID v1.0 are lower (better) than on ASTEROID v0.9 by a factor of 1.4, and do not differ significantly from thresholds on the Randot Circles. Thresholds were roughly log-normally distributed with a mean of 1.54 log_10_ arcsec (35 arcsec) on ASTEROID v1.0 compared to 1.70 log_10_ arcsec (50 arcsec) on ASTEROID v0.9. The standard deviation between observers was the same for both versions, 0.32 log_10_ arcsec, corresponding to a factor of 2 above and below the mean. There was no difference between the versions in their test/retest repeatability, with 95% coefficient of repeatability = 0.46 log_10_ arcsec (a factor of 2.9 or 1.5 octaves) and a Pearson correlation of 0.8 (comparable to other clinical stereotests).

**Conclusion:**

The poorer stereo thresholds previously reported with ASTEROID v0.9 appear to have been due to the relatively large, coarse dots and low density used, rather than to some other aspect of the technology. Employing the small dots and high density used in ASTEROID v1.0, thresholds and test/retest repeatability are similar to other clinical stereotests.

## Introduction

Stereoacuity, which refers to the smallest binocular disparity that a person can perceive,^1^ is important clinically for the assessment of binocular function in disorders such as strabismus and amblyopia.^2,3^ Several clinical stereotests exist for measuring stereoacuity quickly and conveniently, but all have drawbacks, such as limited precision and monocular cues.^2,4^ Monocular cues are particularly problematic, since in theory they mean that a test intended to measure binocular function could be passed by a one-eyed observer.

**Figure 1:**
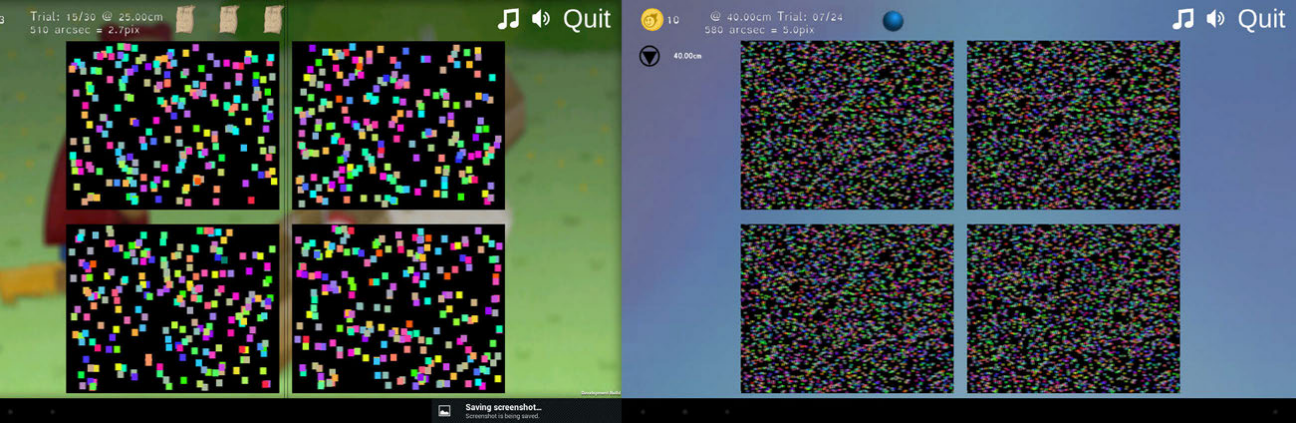
Screenshots from (a) ASTEROID v0.939 and (b) ASTEROID v1.041, showing the different dot size and density of elements in the two versions. In each case, three of the four patches of random dots are flat, while the fourth contains a disparate target which appears to protrude when viewed stereoscopically. The task is to identify which patch contains the target. The disparity of the target is reduced, using a Bayesian staircase, until threshold performance is reached.

Recently, researchers at Newcastle University developed a new stereotest, known as ASTEROID, using an autostereoscopic 3D tablet (www.commander3d.com).^5,6^ As shown in *Figure* 1, users are shown four random-dot patterns and are instructed to select the one that contains a disparate patch appearing to float in front of the tablet screen. The binocular disparity of this patch is reduced following each correct response, and is kept the same or increased following each incorrect response, according to a Bayesian adaptive staircase procedure. An estimate of stereo threshold, corresponding to stereoacuity, is produced after 20 responses have been given. ASTEROID was designed to avoid monocular cues and allow a precise determination of threshold while remaining quick and straightforward enough for use in the clinic. Thus, it may prove especially useful for clinical research.

Stereo thresholds measured with ASTEROID v0.9 are on average around 55 arcsec for non-stereo-blind adults.^6^ This is significantly higher than typically reported for some other stereotests (e.g., 27 arcsec for a lab test presented on a pattern-retarded passive stereoscopic 3D TV viewed through 3D glasses^6^; 20 arcsec on the clinical Frisby stereotest^7^). This could potentially be due to an issue with the autostereo display hardware, e.g., interocular crosstalk, where the left eye sees the image intended for the right eye. However, very similar thresholds were obtained with the same stimuli presented on a Propixx 3D projector, which uses completely different stereoscopic display technology.^8^ We hypothesise that the stimulus is responsible for the observed threshold differences, and in particular the relatively large, sparse dots (18 pixels wide × 20 pixels high, which subtend 18 × 20 arcmin at a viewing distance of 40 cm) used in ASTEROID random-dot stereograms.

The use of sparse, large dots in ASTEROID v0.9 reflected real-world constraints. Avoiding monocular cues on an autostereo device requires dynamic stimuli and a rapid refresh rate (see Serrano-Pedraza *et al*.^9^ for a detailed explanation). The random-dot patterns (*Figure* 1) are therefore constantly changing, although the disparity remains the same until the person responds. Using the optimal disparity on each presentation means that the tablet must compute the stimulus in real time, depending on the previous responses of that individual. Because the 3D tablet is not very computationally powerful, it was not able to compute patterns made up of large numbers of small dots at the necessary refresh rate. Accordingly, ASTEROID v0.9^6^ used relatively fewer and larger dots, which could be computed fast enough to avoid monocular cues.

Secondary issues arise, however, with the use of larger dots. As previously mentioned, the task in ASTEROID, as in other stereotests, is to detect a patch apparently floating in front of a background (*Figure* 1). Because the patch is made up of randomly-placed dots, its edges are not perfectly straight.^6^ Larger dots give the patch a more ragged appearance, which could reduce its salience and lead to higher thresholds. Furthermore, the dot size and density of a random-dot pattern affect its luminance spatial frequency spectrum. Roughly speaking, high spatial frequencies reflect fine detail in the image, while low spatial frequencies reflect structure at coarser scales. Thus, a stimulus with more and smaller dots contains more of its power at high spatial frequencies than does a stimulus with fewer, larger dots, and this in turn can affect thresholds.^3,10–16^

Differences in thresholds between clinical stereotests are not unusual. Thresholds on the TNO stereotest are typically higher than other tests, for example.^17^ Though straightforward comparability between tests is desirable, threshold differences can be taken into account when comparing results across tests. Of greater concern is the fact that patients with poor stereoacuity might fail to perform the task at all, meaning that their stereoacuity may not be measurable using a test with a high threshold.

To resolve these limitations, we sought to employ stimuli with denser, smaller dots. To circumvent the tablet’s computing limitations, stimuli for a fixed set of disparities were pre-computed and stored on the tablet. Since the tablet could only load a relatively small set of stimuli, this meant that we could no longer display the statistically optimal disparity for each trial, instead having the tablet pick the pre-computed image which was nearest to optimal. This approach makes the Bayesian staircase slightly less efficient, which could manifest as higher variability in stereo threshold and thus poorer repeatability.

To investigate these questions, we compared stereo thresholds measured using ASTEROID v0.9 (*Figure* *1a*), ASTEROID v1.0 (*Figure* *1b*), and a standard clinic stereotest, the Randot circles. We hypothesised that the smaller dots and higher density elements of ASTEROID v1.0 would lead to lower (better) stereoacuity thresholds that were similar to those found for Randot circles. We also measured test/retest repeatability to assess whether the loss of statistical efficiency in the new version impacted repeatability.

## Methods

All versions of ASTEROID to date run on a Commander 3D autostereo tablet computer (www.commander3D.com), where each pixel has a physical width of 0.114 mm. Details not discussed below remain as described in Vancleef *et al*.^6^

### ASTEROID stereotest v0.9

In ASTEROID v0.939-v0.95, used here, the dots were 18 pixels wide × 20 pixels high, subtending 14 × 16 arcmin at a viewing distance of 50 cm.^6^ The density of dots was specified such that the dots cover 30% of the stimulus area when not overlapped, meaning that each of the 4 patches contained around 250 individual dots (scattered randomly with overlap). These images were generated by the tablet in real time. The refresh rate could not be controlled precisely, but was around 10 Hz, being limited by the speed with which the tablet could compute the patterns and any other demands placed on it.^9^

### ASTEROID stereotest v1.04

ASTEROID versions v1.0 onwards (*Figure* *1b*) have not been described previously. In this new version, the dots are 6 pixels wide × 6 pixels high (before anti-aliasing, see below), subtending 5 × 5 arcmin at 50 cm. Dot density was specified such that the dots cover 80% of the stimulus area when not overlapped, meaning that each of the four patches contained around 3400 individual dots (scattered randomly with overlap). The use of pre-computed images resulted in a somewhat higher refresh rate. The dots are therefore smaller, denser and faster in v1.0 than in the previous versions.

The need to store pre-computed images impacted the disparities which could be displayed. In this context, it is helpful to distinguish between ‘binocular parallax’, the distance between corresponding features in the on-screen images presented to the left and right eyes, and ’binocular disparity’, the angular separation between the retinal images received to the left and right eyes. The pre-computed images each had a fixed parallax, but the resulting retinal disparity depended on viewing distance.

In ASTEROID, participants select the pattern containing a ’floating’ target from among three distractor patterns depicting a flat surface. To avoid monocular cues, the background of each pattern must have parallax equal and opposite to that of the target.^9^ Thus, we could not use the same distractor patterns for different parallaxes, but had to store separate target and distractor image-files for each parallax. To obtain dynamic stimuli, we had to store multiple image-files for each parallax, which were presented randomly at each screen refresh. We found that randomly shuffling five patterns avoided any percept of repetition. The tablet could not run successfully with more than ~180 pre-loaded images, however, so with 10 image-files per parallax (five different patterns for target and distractor), 18 parallaxes were all that were possible. The parallaxes we chose (0.2, 0.3, 0.4, 0.5, 0.6, 0.7, 0.8, 0.9, 1, 2, 3, 4, 5, 10, 12, 20 pixels) span the range of those typically needed, with additional precision around common thresholds, taking into account the typical viewing distances and the log-normal distribution described below.

The algorithm for obtaining the threshold then had to be slightly modified. On each trial, the tablet computed the desired retinal disparity as described previously.^6^ Based on the current estimate of viewing distance, the tablet then computed the screen parallax, in pixels, corresponding to the desired disparity. In the original version of ASTEROID, v0.9, the tablet then generated a stimulus with this parallax. In the new version, v1.0, it displayed whichever pre-computed parallax was closest in log-space, then converted back from this parallax to find what disparity was actually displayed. This disparity was used to update the probability density function for the stereo threshold, as described in equation (11) of Vancleef *et al*.^6^ As before, the stereo threshold was estimated as the mean of this probability density function at the end of the test.^18^

The use of 18 discrete parallaxes sounds similar to the fixed levels available in clinical stereotests, for example 20, 25, 30, 40, 50, 70, 100, 140, 200, or 400 arcsec on the Wirt circle component of the Randot stereotest. However, in clinical stereotests, the final score is limited to one of these fixed levels. For ASTEROID, the algorithm takes into account all 20 responses, and the final estimate is not limited to the fixed levels, but can take one of roughly a million (2^20^) different values.

**Figure 2:**
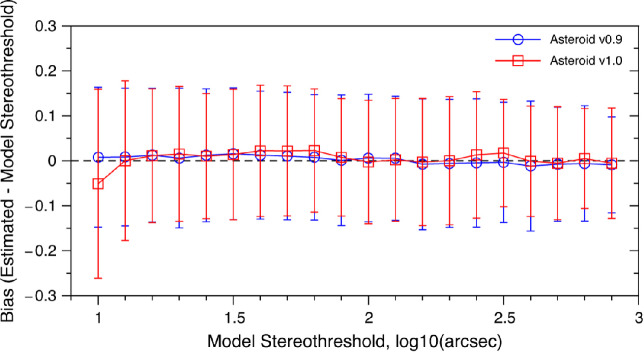
Simulations showing how well the two versions of ASTEROID estimate the threshold of a model observer. In these simulations, we run the test procedure on a simulated observer whose probability of answering correctly on each trial was determined by their psychometric function (equation 7 from^6^ with parameters λ = 0.03, g = 0.25, b = 7.327, A = threshold - 0.112, where threshold was varied. Note that this model observer has a steeper slope b than assumed internally within ASTEROID, where b_true_ = 4.885^5,6^). In each test, the stereo threshold was estimated from 20 trials, i.e., 20 responses from the model observer. We ran 2000 tests on each observer. The true model threshold is plotted on the *x*-axis and the y-axis shows the bias, i.e., the mean difference (in log units) between the true threshold as specified on the *x*-axis and the value returned on the test, both measured in log_10_ arcsec. The error-bars show ± 1 standard deviation. The simulated viewing distance was 50 cm, meaning that the 18 available parallaxes in ASTEROID v1.0 correspond to 9, 14, 19, 24, 28, 33, 38, 42, 47, 94, 141, 188, 235, 470, 564, and 941 arcsec.

The original version of ASTEROID used the statistically optimal disparity recommended by the staircase; the new version used the closest available. We used simulations to assess whether these changes to the algorithm were likely to introduce bias or decrease precision. The simulations were carried out as described by Vancleef *et al*.^6^, but using the modified algorithm of ASTEROID v1.0 (red squares in *Figure* 2) as well as the original v0.9 (blue circles). The results are shown for 20 different model observers whose stereo threshold is plotted on the horizontal axis, ranging from 10 to 800 arcsec. Both versions of the test are virtually unbiased (the mean threshold estimate returned by the test is extremely close to the true value). For example, the penultimate data-point in *Figure* 2 shows that ASTEROID v1.0 can accurately estimate a true threshold of 631 arcsec (2.8 log_10_ arcsec), even though at the simulated viewing distance of 50 cm the closest disparities it could present were 564 and 941 arcsec. By estimating the performance of the simulated observer at these disparities, it is able to make an unbiased estimate of what disparity would elicit a 75% correct performance. Importantly, the precision after 20 responses is the same for both versions, since the standard deviation of the estimates (as shown by the error-bars) are very similar in length.

#### The pre-computed stereograms

**Figure 3:**
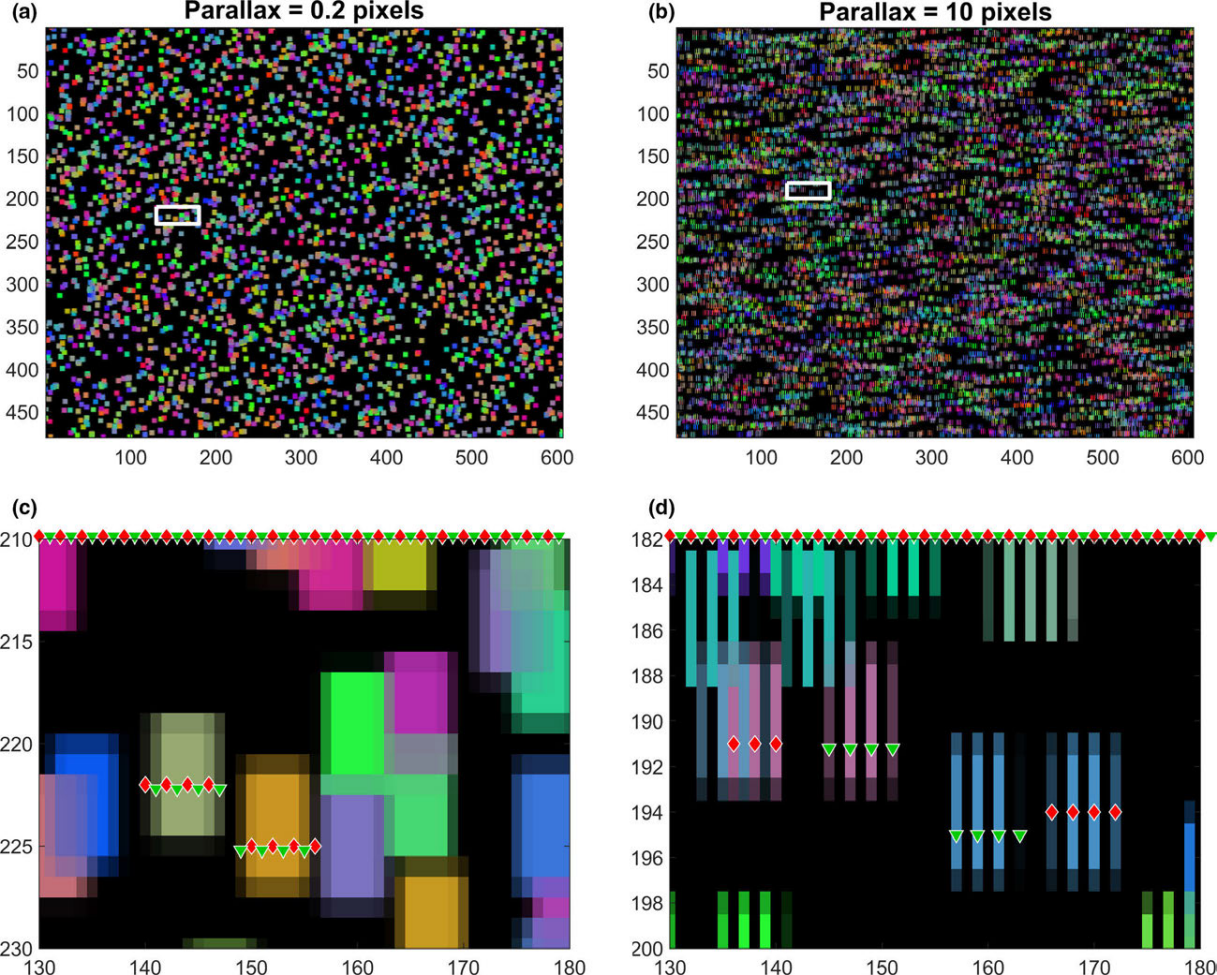
a, b: Example images. The white boxes are not part of the images used in the test, but indicate the parts of the image which are shown in c, d. c, d: Zooming in to show individual dots. These are column-interleaved stereo images. With the tablet held correctly, the parallax barrier means that the columns marked with red diamonds are visible to the left eye only while the columns marked with green triangles are visible to the right eye only.

The stereograms need to be generated with care to provide sub-pixel disparities while avoiding monocular cues due to the column-interleaved display.^9^ We therefore describe in detail how this is achieved for readers who may wish to reproduce this. *Figure* 3 shows two example stimuli from ASTEROID v1.04. Both contain a disparate target at the centre of the image. The images are column-interleaved stereograms which appear in 3D when viewed on the tablet due to its parallax barrier. The image destined for the left eye is shown on even pixel columns, marked with red diamonds in *Figure* *3c*,*d*, while the right image is on odd columns, marked with green triangles. The white boxes in *Figure* *3a*,*b* are displayed in the test, but mark the region of each image which is zoomed in the panel below. Here, we can see the individual dots. We marked the left and right-eye pixels on two dots in each image. In each case, the left-hand dot is in the background and so is presented behind the screen plane (this is the beige dot in *Figure* *3c* and the pink dot in *Figure* *3d*). The left-eye image of the background dot therefore appears to the left of the right-eye image. This is clearest in the pink dot marked in *Figure* *3d*. The pink vertical lines in even pixel columns 136, 138 and 140, marked with red diamonds, represent the dot in the left eye, while the pink vertical lines in odd columns 145, 147, 149 and 151, marked with green triangles, represent the corresponding dot in the right eye. The gold dot in *Figure* *3c* and the blue dot in *Figure* *3d* are in the disparate target, and so have equal and opposite parallax. For the blue dot in *Figure* *3d*, the left-eye image in pixel columns 166, 168, 170 and 172 are to the right of the right-eye image in columns 157, 159, 161 and (faintly) 163.

In the stereogram with parallax 10 pixels, *Figure* *3b*,*d*, the left- and right-eye images of each dot are clearly distinct. In the stereogram with parallax 0.2 pixels, *Figure* *3a*,*c*, they overlap. However, in the left-eye image of the background, the beige dot (columns 140, 142, 144, 146) still lies to the left of the corresponding right-eye dot (columns 141, 143, 145, 147), indicating a depth behind the screen. For the gold dot in the target, the left-eye pixels (150, 152, 154 and (faintly) 156) are to the right of the right-eye (149, 151, 153, 155), indicating depth in front of the screen.

In the Introduction, we described the dots as 6 × 6 pixels, yet *Figure* 3 shows some dots extending over more than 6 pixels. This is due to the anti-aliasing used to position each dot with sub-pixel precision. For example, to represent a dot whose lower edge is at *y* = 221.3, the pixel at *y* = 221 was assigned 0.7 of the dot's intended luminance in each colour channel; the five pixels at *y* = 222–226 were given the full luminance, while the pixel at *y* = 227 is given 0.3 of the luminance. Thus, dots extended over either 6 or 7 pixels vertically. A similar manipulation was applied horizontally, after shifting the dot positions in left and right half-images to achieve the desired parallax. Additionally, as seen in *Figure* 3, the column-interleaving means that gaps extend vertically across each dot. Thus, dots horizontally spanned either 6 or 7 pixels on the tablet, meaning 3 or 4 pixels in each eye’s individual image.

### Participants

Data were collected in Newcastle, UK, and Madrid, Spain, from adult participants aged between 18 and 30 years. All participants in both locations gave informed written consent to participate and the research followed the tenets of the Declaration of Helsinki. Participants were not screened for their vision or stereopsis, since the aim here was to investigate the effect of dot size within each subject, rather than to obtain normative values.

In Newcastle, 45 participants were recruited from students of Newcastle University and Newcastle University Institute of Neuroscience Research Volunteer pool. One participant, with a history of amblyopia, could not perceive stereo depth in either test and so was excluded from analysis, leaving 44 participants (20 men, 24 women). In Madrid, 31 participants (18 men, 13 women) were recruited from students of Universidad Complutense de Madrid. The results report combined data from 75 participants (38 men, 37 women). In the subsequent *Figures*, we use different symbols to indicate the test location, but as the results did not differ between sites, the data were pooled for analysis.

### Protocol

Each participant completed both versions of the ASTEROID test twice. The ’large dots’ version was v0.939, v0.940 or v0.95. The ’small dots’ version was v1.041. The sequence was either (1) large, small, large, small or (2) small, large, small, large, chosen randomly.

**Figure 4:**
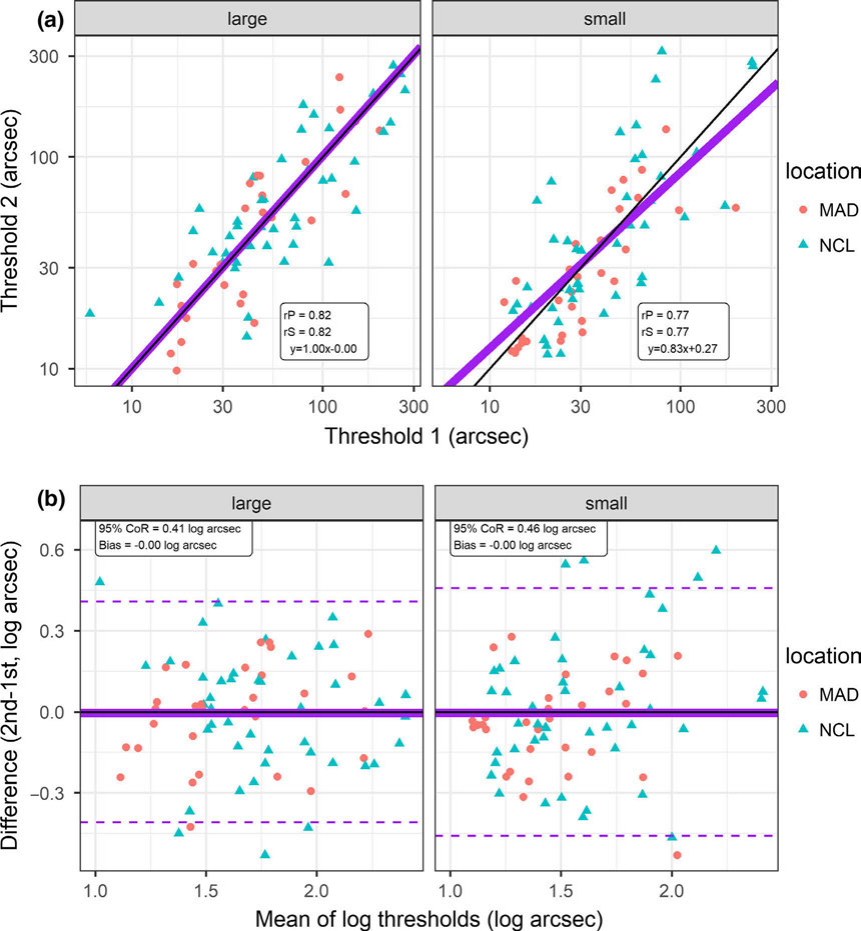
Test/retest repeatability of ASTEROID with large dots (versions 0.940 and 0.939, left panels) and with small dots (version 1.041, right panels); *n* = 75. Colours/symbols indicate test location: Madrid (MAD) or Newcastle (NCL). a: Scatterplots showing stereo threshold measured on second test, θ_2_, vs threshold on first test, θ_1_. Black line shows identity; purple line shows Model II major axis regression (i.e., accounting for the fact that both variables are subject to error). Text box shows regression equation (note that *x*, *y* are log values) plus Pearson (rP) and Spearman (rS) correlation coefficients. b: Bland-Altman plots showing difference in log arcsec (log_10_θ_2_ − log_10_θ_1_) plotted against the mean (log_10_θ_2_ + log_10_θ_1_)/2. A log transform is used since log thresholds are closer to normally distributed than the original log thresholds 44. Horizontal black line shows 0 difference; solid purple line shows mean of observed differences, or bias; dashed purple lines show the limits of agreement, ±1.96 standard deviations of the observed differences, or 95% coefficient of repeatability; both are given in the text box. Since the bias did not differ significantly from 0, we centred these lines around 0 rather than the bias. *N* = 75 participants.

**Figure 5:**
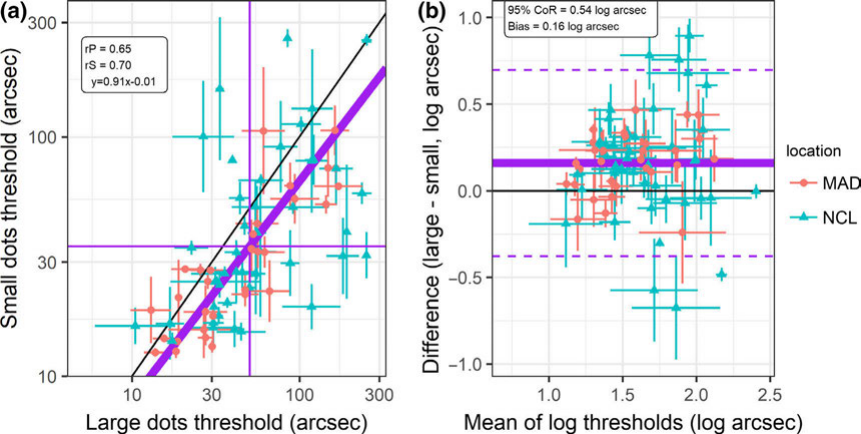
a: Scatterplot, and b: Bland-Altman comparison between thresholds obtained with large vs small dots (*n* = 75). In (a), the horizontal and vertical axes show the geometric mean of all thresholds obtained for the respective dot size. Most participants only took two thresholds with each dot size, but five took 4 and author ZYW took 12. Black line marks identity, thick purple line the major axis Model II regression, and vertical/horizontal thin purple lines the average threshold on each test. Textbox reports Pearson (rP) and Spearman (rS) correlation coefficients along with regression equation (*x*, *y* are log thresholds). The error bars link the two thresholds recorded for each participant (or ±1 S.E.M. for participants who recorded >2 thresholds). In b, the vertical axis shows the difference between the mean of all log thresholds obtained for a given dot size, while the horizontal axis shows the mean of these means. The error bars in (b) are the same horizontally and vertically, and are equal to (s_small_^2^ + s_large_^2^)^0.5^, where s_large_ and s_small_ are the lengths of the horizontal and vertical error bars in (a), respectively. Horizontal purple line shows mean of the differences (bias); dashed lines show ± 1.96 standard deviations about this, i.e., the 95% limits of agreement; text box gives values for these. The bias of 0.16 log_10_ arcsec corresponds to a factor of 1.4 in arcsec. MAD = Madrid, NCL = Newcastle.

Five Newcastle participants did each test a further two times and Newcastle author ZYW did each test 12 times. To compare versions (*Figure* 5), we used all data collected; we took the mean of the log-thresholds, since the number of repetitions does not bias the estimate of mean difference.^19^ For the repeatability assessment (*Figure* 4), we used only the first two measurements for all participants.

The tablet was put in a case containing an integral stand and placed at a distance of 50 cm throughout the four tests. Participants used a chin rest to avoid head movements. The tablet assumed a default viewing distance when converting from screen parallax to angular disparity: this was 25 cm for the large-dot v0.9 and 40 cm for the small-dot v1.0. Before analysis, all data were therefore corrected for viewing distance by multiplying the original threshold estimate by (tablet's default viewing distance)/(actual viewing distance). For example, if the tablet reported a stereoacuity of 30 arcsec while running ASTEROID v0.9, where the default viewing distance is 25 cm, we corrected this to 30 * 25/50 = 15 arcsec to account for the fact that the actual viewing distance was twice as far as assumed by the tablet.

In Madrid, participants also completed a standard clinical stereotest, the Randot Circles (Stereo Optical Company, www.stereooptical.com), at its standard viewing distance of 40 cm. Newcastle participants did not perform this comparison.

### Analysis

#### Normality transform and note on units

Clinicians generally report stereoacuity in arcsec. Empirically, stereoacuity is found to be distributed roughly log-normally. That is, the logarithm of stereoacuity is distributed normally, whereas stereoacuity in arcsec is skewed, with a long tail of high values. For this reason, all analysis in this paper was conducted on log-stereoacuity. For example, when averaging results from different observers, we take the mean of the log-stereoacuities in log_10_ arcsec, and then raise to 10 to the power of this result to express the average in the more familiar stereoacuity units of arcsec. We refer to this process as the ’average’. Thus, if we report ’average threshold = 50 arcsec’, we mean that we have first computed the arithmetic mean of the log_10_ thresholds and then converted to arcsec. To see the difference, consider values of 100, 200, 400, 800 arcsec. The arithmetic mean of these thresholds is 375 arcsec, but this gives too much weight to the larger values, where the error (in arcsec) is larger. The mean of the log-thresholds is 2.45 log_10_ arcsec or 283 arcsec. Correlations and all other metrics are similarly computed on log-thresholds.

Confidence intervals cannot meaningfully be converted to arcsec, but have to be expressed as factors, because they are wider for people who score worse. For example, a 95% coefficient of repeatability of ±0.5 log_10_ arcsec corresponds to a factor of 3.2 in arcsec. If Person A is initially measured as having a log-stereoacuity of 1.5 log_10_ arcsec (=10^1.5^ or 32 arcsec), we can be 95% confident that a subsequent measurement will lie between 1.0 and 2.0 log_10_ arcsec (10–100 arcsec: i.e., from 1/3.2 times to 3.2 times their original score of 32 arcsec). Similarly, if Person B initially scores 2.7 log_10_ arcsec (=500 arcsec), we can be 95% confident that a subsequent measurement will lie between 2.2 and 3.2 log_10_ arcsec (160 to 1600 arcsec, i.e., from 500/3.2 to 500*3.2). The same confidence interval on log-thresholds (0.5 log_10_ arcsec) corresponds to a range of 90 arcsec for Person A, but to a range of 1440 arcsec for Person B.

In the clinical literature, factors are sometimes also reported as octaves. 1 octave corresponds to a factor of 2; 2 octaves to a factor of 4; and in general *x* octaves to a factor of 2^x^.

#### Statistical tests

Different stereotests (e.g., ASTEROID v0.9 vs v1.0, or vs Randot Circles) were compared using a paired *t*-test, by a Bland-Altman analysis producing the 95% coefficient of repeatability^20^ and also by the Pearson correlation coefficient between test and retest scores. Test/retest repeatability was evaluated by the same metrics. To compare repeatability between versions, we used an *F*-test to compare the variance of the test-retest differences.

#### Code

The analysis was done in RStudio (www.rstudio.com) using the following packages: R [Version 3.6.0^21^] and the R-packages cowplot version 1.0.0,^22^ data.table version 1.12.2,^23^ DescTools version 0.99.30,^24^ dplyr version 0.8.3,^25^ ggplot2 version 3.1.1,^26^ lmodel2 version 1.7.3,^27^ lubridate version 1.7.4,^28^ papaja version 0.1.0.984,^29^ stringr version 1.4.0^30^ and tidyr version 1.0.0.^31^ Analysis code and data files are available at https://doi.org/10.25405/data.ncl.11815845.

## Results

### Test-retest repeatability

*Figure* 4 shows scatterplots (top row) and Bland-Altman plots (bottom) comparing the thresholds obtained with the first and second measurements on each test. There is no evidence for a practice effect: the thresholds on first and second tests did not differ significantly (*p* > 0.05, paired *t*-test on log thresholds).

For large dots, the results agree with previous studies using ASTEROID v0.9.^6,8^ The test/retest correlation is high: (*r* = 0.82, *p* < 10^−6^). The 95% coefficient of repeatability^20^ is ±0.41 log_10_ arcsec, corresponding to a factor of 2.6 in arcsec. Both of these values agree closely with previous studies of large-dot versions of ASTEROID, summarised in *Table* 1.

**Table 1:** Typical average values in a healthy young-adult population, and test/retest repeatability, of different ASTEROID versions as reported in this and previous studies. Note that the population mean given for v1.041 is for all 75 participants, and is thus slightly different from the value shown in *Figure* 6 for the 31 participants who also completed the Randot Circles

ASTEROID version	Study	Distribution	Test/retest repeatability			
		Geometric mean threshold (mean ± S.D. for log thresholds)	Correlation coefficients sample (95% confidence interval)		95% coefficient of repeatability/ coefficient of repeatability 20	
			Pearson	Spearman	± log_10_ arcsec	As a factor
v0.9x	Vancleef *et al*.^6^	57 arcsec (1.75 ± 0.34 log_10_arcsec)	0.80	0.63	0.64	4.3
v0.9x	McCaslin *et al*.^8^	59 arcsec (1.77 ± 0.34 log_10_arcsec)	0.82	0.63	0.37	2.3
v0.9x	Present study	50 arcsec (1.70 ± 0.33 log_10_arcsec)	0.83 (0.73–0. 88)	0.82 (0.73–0.89)	0.41	2.6
v1.041	Present study	35 arcsec (1.54 ± 0.32 log_10_arcsec)	0.77 (0.65–0.85)	0.77 (0.65–0.85)	0.46	2.9

For the new small-dot version, test/retest repeatability is similar to the older large-dot version. The correlation is *r* = 0.77 (*p* < 10^−6^) and the 95% coefficient of repeatability is ± 0.46 log_10_ arcsec, corresponding to a factor of 2.9 in arcsec. There was no significant difference in repeatability between the large- and small-dot versions of the tests (*p* = 0.33, *F*-test for whether the variance of the inter-session differences is the same for large vs small dots). Thus, we find that the new small-dot version has similar test/retest repeatability as the original large-dot version.

### Stereoacuity with large vs small dots

*Figure* 5 compares stereoacuity as measured on the old and new versions of ASTEROID. Stereoacuities measured on the two versions are strongly correlated (*r* = 0.65, *p* < 10^−6^). However, thresholds are higher with the large dots. Taking the mean of log-thresholds across subjects, the average is 50 arcsec with large dots as against 35 arcsec with small: participants score higher (that is, worse) with large dots by a factor of 1.4. This difference is highly significant (*t* = 5.1, *p* = 3×10^−6^).

### Comparing ASTEROID with Randot Circles stereoacuity

**Figure 6:**
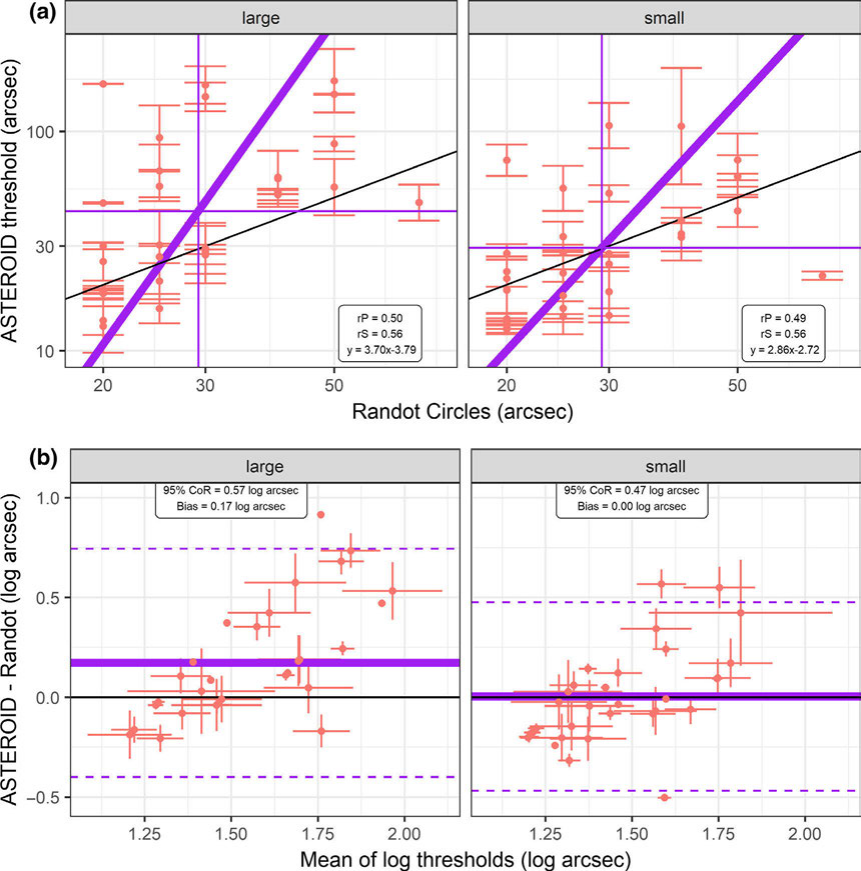
(a) Scatterplot and (b) Bland-Altman analysis comparing stereo thresholds on Randot vs ASTEROID: (left) large-dot ASTEROID v0.9, (right) small-dot ASTEROID v1.0. Data for ASTEROID are the average of the two measurements; error bars are the difference between the two measurements. Only one measurement was taken on Randot, so no error bars are plotted. Available thresholds on Randot Circles are 20, 25, 30, 40, 50, 70, 100, 140, 200, 400 arcsec. These data are from the 31 participants in Madrid (MAD). (a) Black line shows the identity; the purple line shows the major axis Model II regression; the purple lines show the population average (mean log_10_ threshold) for each test. The text box reports the Pearson (rP) and Spearman (rS) correlation coefficients and the regression equation (*x*, *y* are log thresholds). (b) Bland-Altman analysis. The vertical axis shows the difference between the log thresholds obtained on ASTEROID and those obtained on Randot, while the horizontal axis shows the mean. Horizontal purple line shows mean of the differences (bias); dashed lines show ± 1.96 standard deviations about this, i.e., the 95% limits of agreement; text box gives their values.

The 31 participants in Madrid were also tested with the Randot Circles stereotest. Thresholds with large-dot ASTEROID v0.9 are significantly higher than with Randot Circles (43.4 vs 29.2 arcsec; *t* = 3.29, *p* = 0.003). However, with small-dot ASTEROID v1.0, stereo thresholds were not significantly different from those for Randot Circles (29.5 vs 29.2 arcsec; *t* = 0.10, *p* = 0.92). The 95% confidence interval on the mean between the log threshold spans −0.08 to 0.09 log arcsec. This means that while ASTEROID v1.0 and Randot Circles are in good agreement, we cannot exclude the possibility that stereo thresholds measured on ASTEROID v1.0 are actually systematically smaller than thresholds measured with the Randot Circles by a factor of as little as 0.82, or systematically larger by a factor of as large as 1.24 (*Figure* 6).

## Discussion

In this paper, we have compared stereo thresholds measured on earlier versions of ASTEROID (v0.939–0.950) with those measured on a later version (v1.041). Both versions used a four-alternative disparity detection task in a dynamic random-dot stereogram, but whereas the earlier versions used large, sparse dots, the new versions used small, dense dots which were also more rapidly refreshed on the screen. As predicted, we find that stereo thresholds are smaller when measured with a stereogram consisting of finer, denser dots. The change is a factor of 1.4, very similar to the difference previously observed between ASTEROID v0.9 and other stereotests. This suggests that, as we suspected, this difference between ASTEROID and other stereotests was due to the large, sparse dots used in ASTEROID v0.9. Since our purpose here was to establish typical scores for the newer ASTEROID, we have not sought to disentangle the contributions made by the smaller dot size, the increased density and the increased refresh rate. We simply conclude that the smaller, denser, faster dots together have brought ASTEROID thresholds into agreement with those typically measured on other stereotests.

### Dot size and visual acuity

The dot width in ASTEROID v0.9 was 18 arcmin, much larger than usual in random-dot-style stereograms, where typical dot widths are 2–6 arcmin.^10,32–38^ In ASTEROID v1.0, dot width was reduced to 6 arcmin. Another digital stereotest by Tittes *et al*.^39^ used dots generated from a log-Gabor function with a spatial frequency of 0.5 cycles per degree (period 120 arcmin), with a central bright blob and darkened surround. They are said to have an effective diameter of 10 arc min, but this presumably refers to the central bright blob, with the surrounding dark region being much larger. With this test, the median stereoacuity was 51 arcsec, similar to ASTEROID results for large dots. Tittes *et al*. found that many control subjects obtained lower (better) thresholds on the TNO stereotest than on their digital stereotest, which they attributed to the finer spatial resolution of the TNO stimulus, in agreement with our conclusion.

Importantly, whereas our paper includes only healthy controls, Tittes *et al*.^39^ also studied patients with amblyopia. They found that in contrast to controls, these patients often obtained better thresholds on the digital test than on TNO. They suggest this is because the larger dots were easier for these patients to see with both eyes. The overall conclusion seems to be that although stimuli with coarser spatial frequencies will not allow acute observers to display their best possible stereopsis, they may facilitate stereopsis in observers with limited visual acuity. This could be a reason to prefer ASTEROID v0.9 in some circumstances.

### Test/retest repeatability

In ASTEROID v0.9, the disparity displayed on each trial is chosen to be the most statistically efficient.^6^ In ASTEROID v1.0, the disparity displayed is the closest to this value out of the 18 available. The staircase is therefore theoretically slightly less efficient. However, simulations indicate that this makes no difference to the precision after 20 responses (*Figure* 2), and empirically, test/retest repeatability was not statistically different between the two versions (*Figure* *4b*). This is probably because repeatability is constrained mainly by the small number of responses, so any decrease in efficiency due to the limited number of available parallaxes has negligible effect. This suggests there is no practical disadvantage to using pre-computed images.

The 95% coefficient of repeatability of 0.46 log_10_ arcsec, corresponding to a factor of 2.9, is extremely high. It means that a patient who scored 400 arcsec on the initial test might score as low as 138 arcsec or as high as 1160 arcsec on a subsequent retest without any real change in their vision, simply due to the poor repeatability of the test. This is unfortunately a feature of all clinical stereotests, which attempt to estimate stereoacuity from just a few test disparities. For example, Adams *et al*.^40^ report 95% coefficients of repeatability corresponding to factors of 3.9 for the Preschool Randot, 1.7 for the near Frisby, 4.8 for the Frisby-Davis 2, and 2.9 for the Distance Randot. Thus, ASTEROID is similar to other clinical stereotests in this regard.

Ultimately this reflects the fact that, even in people with ’normal’ stereoscopic vision, disparity appears to be subject to a relatively large amount of noise. We can quantify this noise as having an S.D. of around 0.3 log_10_ arcsec. It is in log units because, in an example of the general Weber-Fechner law for perception, the signal available to the visual system is closer to the logarithm of disparity than to disparity itself. Examining the slope of psychometric functions enables us to estimate that this log-disparity signal is affected by noise with an S.D. of 0.3 log_10_ arcsec (see equation 5 of Vancleef *et al*.^6^and appendix A1 of Serrano-Pedraza *et al*.^5^, with σ = 1.1 log_10_ arcsec; compare with *figure 4*^5^). This means that any given disparity is represented within the visual system as a noisy signal ranging from 25% to 400% of the true disparity. This range is derived by taking ±2 S.D. as the 95% CI and converting logs to factors. According to standard models, a person detects a disparity when this noisy signal exceeds a fixed threshold. Thus, over a wide range of disparities, performance is probabilistic: a person might detect a disparity on one trial but not another, depending on the noise. Only when the disparity is four times higher than threshold will the person detect it reliably. Accordingly, for disparities closer to threshold, it takes several repetitions to establish whether that disparity is in fact above or below threshold.

This is why it is difficult to establish stereoacuity precisely from the few responses feasible within a clinical setting, and why clinical stereotests have such wide confidence intervals. If it is important to measure stereoacuity precisely, for example to be sure whether treatment has brought about improvement, then several threshold measurements should be made and log-averaged as described above. The 95% coefficient of repeatability in arcsec will be reduced by 1/√*N*, where *N* is the number of measurements. For example, averages of 3 thresholds should agree to within a factor of 1.8 (10^0.46/√3^).

### Anti-aliasing artefacts

ASTEROID v1.0 used anti-aliasing to achieve sub-pixel disparities, whereas versions v0.939 and v0.94 used temporal dithering.^6^ As we noted previously,^6^ precise anti-aliasing requires accurate luminance linearisation, but we did not attempt this in ASTEROID, since it would have to be done for each individual tablet and users might alter the display settings. Since 1 pixel is around 60 arcsec in ASTEROID, it is possible that differences in sub-pixel disparities contributed to the difference we observed in stereoacuity measured with the two versions. Arguing against this, a recent study^8^ found close agreement between ASTEROID v0.9 and the same stimuli presented on a PROPixx 3D projector (www.vpixx.com) where luminance was linearised. Furthermore, luminance non-linearities would not be expected to bias disparity, but rather to introduce a form of spatial dithering. For example, suppose that we wish to depict a white dot beginning at *x* = 10.7 pixels in the left eye. We would intend to display luminances of 0.3 times the maximum for pixel 10, 1.0 for pixels 11–15, and 0.7 for pixel 16. Suppose that the display nonlinearity means that luminances below/above half the maximum are displayed less/more than intended. The dot will effectively be shifted to the right. Now suppose the dot has a disparity of 0.5 pixels, so the dot begins at *x* = 11.2 pixels in the right eye. We would depict this with luminances of 0.8 for pixel 11, 1 for pixels 12–16, 0.2 for pixel 17. The nonlinearity would mean that this dot would effectively be shifted to the left. Overall, the disparity would be less than intended. However, another dot might be positioned at *x* = 12.2 pixels in the left eye and 12.7 pixels in the right eye. By the same argument as above, the nonlinearity would effectively shift the dot left in the left eye and right in the right eye, increasing the disparity above that intended. This is a form of spatial dithering in which on average the dots have the intended disparity. Thus, the two versions of ASTEROID may both effectively achieve sub-pixel disparities by dithering: v0.9 by temporal dithering and v1.0 by spatial dithering. The good agreement with the PROPixx system suggests that this dithering is successful.

### Comparison with Randot Circles clinical stereotest

Randot Circles, also known as Wirt Circles, is a clinical stereotest designed to measure fine stereopsis in adults. On each trial, the task is to detect which of three black circles is stereoscopically closer. Since the circles are defined by luminance and thus monocularly visible, this is a contour rather than a cyclopean stereotest like ASTEROID.^2,41^ The monocular cues provided by the displacement of the circles means that stereoblind patients can ’pass’ the Randot Circles down to 140 arcsec or better (lower).^4,42^ However, Randot Circles permits scores as low as 20 arcsec, whereas the Preschool Randot test, which is a cyclopean stereotest, has a lowest (best) possible score of 40 arcsec, meaning that most adults obtain the best possible score. Randot Circles is one of the most widely used stereotests in eye clinics, especially in North America.^43^ Accordingly, it is useful to compare scores on ASTEROID with scores on this widely-used clinical stereotest.

A recent study^8^ found a correlation of *r* = 0.54 between Randot Circles and large-dot ASTEROID (v0.931, which used 28-pixel dots) in *n* = 39 participants, including children and stereo-impaired participants. This is very similar to what we see in the present study: *r* = 0.50 between Randot Circles and large-dot ASTEROID (v0.95, *n* = 31) and *r* = 0.49 between Randot Circles and small-dot ASTEROID (v1.041, *n* = 31).

The previous, large-dot ASTEROID v0.9 produced higher stereo thresholds than Randot Circles. However, with the revised small-dot ASTEROID v1.0, thresholds match closely. In the 31 participants who did all three tests, the mean score was 1.47 log_10_ arcsec (30 arcsec) on both Randot Circles and small-dot ASTEROID v1.0, compared to 1.64 log_10_ arcsec (43 arcsec) with large-dot ASTEROID v0.9.

Perfect agreement would not necessarily be expected, given differences in stimuli and task design. For an observer with fixed internal properties (effective noise level ξ, detection threshold A, lapse rate λ; see Vancleef *et al*.^6^ for details and underpinning assumptions), the disparity Δ corresponding to performance of Θ correct on a task where chance is *g* is:

$$ {\log}_{10}\theta =A-\xi \ln \left(\frac{1-\lambda -\Theta}{\Theta -g}\right) $$

ASTEROID uses a 4-alternative task, *g* = 1/4, and defines threshold as a performance level of Θ = 75% correct, so for this observer we would expect to measure:

$$ {\log}_{10}\kern0.166667em {\uptheta}_{\mathrm{AST}}=A-\upxi \kern0.166667em \mathrm{In}\kern0.166667em \left(0.50-2.0\lambda \right) $$

Randot Circles uses a 3-alternative task, *g* = 1/3, and its protocol produces estimates corresponding to performance levels varying from 65% for poor observers to >80% for good ones.^17^ Thus, for the same observer and assuming Θ = 80%, we would expect to measure a slightly higher threshold:

$$ {\log}_{10}\kern0.166667em {\uptheta}_{\mathrm{Ran}}=A-\upxi \kern0.166667em \mathrm{In}\kern0.166667em \left(0.42-2.1\uplambda \right) $$

solely due to differences in the task structure and ignoring any differences in *A* and ξ which might exist due to the stimuli. The noise level ξ = 1/*b* where *b* is the slope of the psychometric function, which we have found^5^ is typically *b* = 6.17/log_10_ arcsec, yielding ξ = 0.16 log_10_ arcsec. For such an observer, Randot Circles thresholds would be around 0.033 log_10_ arcsec (a factor of 1.08 in arcsec) higher than ASTEROID, due solely to differences in the task.

Furthermore, Randot Circles uses static stereograms with monocularly-visible contours, while ASTEROID uses dynamic random-dot stereograms with a cyclopean target lacking monocularly-visible contours. In principle, these stimulus differences might produce internal signals with different effective noise and detection thresholds. Any differences due to stimuli might either add to, or tend to cancel, differences due to the task.

Finally, the lowest (best) possible score on Randot Circles is 20 arcsec. Some participants who scored 20 arcsec probably would have scored lower still if that had been available, bringing down the average score on Randot Circles to lower than the value on ASTEROID v1.0.

Thus, it is likely that larger studies will reveal small differences between ASTEROID v1.0 and Randot Circles. What is clear is that using smaller, denser dots in ASTEROID results in thresholds much closer to those on the Randot Circles and other clinical stereotests.

## Conclusion

The higher (worse) stereo thresholds measured with ASTEROID v0.9 compared to other stereotests reflect the relatively large, coarse dots and low density used in version 0.9. With the small dots and high density used in ASTEROID v1.0, thresholds are very similar to those measured on other stereotests such as the Randot Circles (population average ~30 arcsec). Test/retest repeatability is unchanged, with the limits of repeatability remaining around a factor of 2.9 (0.4 log_10_ arcsec or 1.5 octaves) and the Pearson correlation between test and retest being ~0.8. The new ASTEROID v1.0, with small dense dots, may be preferred to the older v0.9 with large sparse dots, as it is simpler to compare with existing stereotests and the previous literature. However, participants with low acuity may not be able to resolve the smaller dot pattern in v1.0. For these patients, v0.9 with larger dots may give a more accurate representation of their stereoacuity.

## Disclosures

JCAR does consultancy work for Magic Leap. The ASTEROID stereotest has been licensed to a company (no financial implications for any of the authors).

## References

[1] Howard IP & Rogers BJ. *Seeing in Depth, Volume 2: Depth perception*. I. Porteous: Ontario, 2002.

[2] Read JCA. Stereo vision and strabismus. *Eye Lond Engl* 2015; 29: 214–224. 10.1038/eye.2014.279 PMC4330289 25475234

[3] Westheimer G. Clinical evaluation of stereopsis. *Vis Res* 2013; 90: 38–42. 23092634 10.1016/j.visres.2012.10.005

[4] Chopin A, Chan SW, Guellai B, Bavelier D & Levi DM. Binocular non-stereoscopic cues can deceive clinical tests of stereopsis. *Sci Rep* 2019; 9: 5789. 30962466 10.1038/s41598-019-42149-2 PMC6453951

[5] Serrano-Pedraza I, Vancleef K, Herbert W, Goodship N, Woodhouse M & Read JCA. Efficient estimation of stereo thresholds: what slope should be assumed for the psychometric function? *PLoS One* 2020; 15: e0226822. 31895925 10.1371/journal.pone.0226822 PMC6939937

[6] Vancleef K, Serrano-Pedraza I, Sharp C *et al*. ASTEROID: a new clinical stereotest on an autostereo 3D tablet. *Transl Vis Sci Technol* 2019; 8: 25. 10.1167/tvst.8.1.25 PMC6396686 30834173

[7] Bohr I & Read JCA. Stereoacuity with frisby and revised FD2 stereo tests. *PLoS One* 2013; 8: e82999. 24349416 10.1371/journal.pone.0082999 PMC3861460

[8] McCaslin A, Read JCA, Vancleef K & Port N. Stereotest comparison: efficacy, reliability, and variability of a new glasses-free stereotest. *Transl Vis Sci Technol* 2020; 9: 29. 10.1167/tvst.9.9.29 PMC7442860 32879785

[9] Serrano-Pedraza I, Vancleef K & Read JCA. Avoiding monocular artifacts in clinical stereotests presented on column-interleaved digital stereoscopic displays. *J Vis* 2016; 16: 13. 10.1167/16.14.13 PMC5114011 27846341

[10] Gantz L & Bedell HE. Variation of stereothreshold with random-dot stereogram density. *Optom Vis Sci* 2011; 88: 1066–1071. 21642889 10.1097/OPX.0b013e3182217487 PMC3163004

[11] Hess RF, Hong Liu C & Wang Y-Z. Luminance spatial scale and local stereo-sensitivity. *Vision Res* 2002; 42: 331–342. 11809485 10.1016/s0042-6989(01)00285-1

[12] Schor CM, Wood IC & Ogawa J. Spatial tuning of static and dynamic local stereopsis. *Vis Res* 1984; 24: 573–578. 6740978 10.1016/0042-6989(84)90111-1

[13] Schor CM & Wood I. Disparity range for local stereopsis as a function of luminance spatial frequency. *Vis Res* 1983; 23: 1649–1654. 6666067 10.1016/0042-6989(83)90179-7

[14] Westheimer G & McKee SP. Stereoscopic acuity with defocused and spatially filtered retinal images. *J Opt Soc Am* 1980; 70: 772–778.

[15] Simons K. Stereoacuity norms in young children. *Arch Ophthalmol* 1981; 99: 439–445. 7213162 10.1001/archopht.1981.03930010441010

[16] Banks MS, Gepshtein S & Landy MS. Why is spatial stereoresolution so low? *J Neurosci* 2004; 24: 2077–2089. 14999059 10.1523/JNEUROSCI.3852-02.2004 PMC6730432

[17] Vancleef K, Read JCA, Herbert W, Goodship N, Woodhouse M & Serrano-Pedraza I. Overestimation of stereo thresholds by the TNO stereotest is not due to global stereopsis. *Ophthalmic Physiol Opt* 2017; 37: 507–520. 28337792 10.1111/opo.12371 PMC5516234

[18] King-Smith PE, Grigsby SS, Vingrys AJ, Benes SC & Supowit A. Efficient and unbiased modifications of the QUEST threshold method: theory, simulations, experimental evaluation and practical implementation. *Vision Res* 1994; 34: 885–912. 10.1016/0042-6989(94)90039-6 8160402

[19] Bland JM & Altman DG. Measuring agreement in method comparison studies. *Stat Methods Med Res* 1999; 8: 135–160. 10501650 10.1177/096228029900800204

[20] Bland JM & Altman DG. Statistical methods for assessing agreement between two methods of clinical measurement. *Lancet* 1986; 1: 307–310. 2868172

[21] R Core Team. *R: A Language and Environment for Statistical Computing*. R Foundation for Statistical Computing: Vienna, 2019.

[22] Wilke CO. *cowplot: Streamlined Plot Theme and Plot Annotations for “ggplot2.”* https://CRAN.R-project.org/package=cowplot (Accessed 1/8/2020).

[23] Dowle M & Srinivasan A. data.table: Extension of ‘data.frame’, 2019, https://CRAN.R-project.org/package=data.table (Accessed 1/8/20).

[24] Signorell A. DescTools Tools for Descriptive Statistics [Internet], 2019. https://cran.r-project.org/package=DescTools (Accessed 1/8/20).

[25] Wickham H, François R, Henry L & Müller K. dplyr: A Grammar of Data Manipulation, 2019. https://CRAN.R-project.org/package=dplyr (Accessed 1/8/20).

[26] Wickham H. *ggplot2: Elegant Graphics for Data Analysis*. Springer-Verlag: New York, NY, 2016.

[27] Legendre P. lmodel2: Model II Regression, 2018. https://CRAN.R-project.org/package=lmodel2 (Accessed 1/8/20)

[28] Grolemund G & Wickham H. Dates and times made easy with {lubridate}. *J Stat Softw* 2011; 40: 1–25.

[29] Aust F & Barth M. papaja: Create APA manuscripts with R Markdown, 2018. https://github.com/crsh/papaja (Accessed 1/8/20).

[30] Wickham H. stringr: Simple, Consistent Wrappers for Common String Operations, 2019. https://CRAN.R-project.org/package=stringr (Accessed 1/8/20).

[31] Wickham H & Henry L. tidyr: Tidy Messy Data, 2019. https://CRAN.R-project.org/package=tidyr (Accessed 1/8/20).

[32] Glennerster A. dmax for stereopsis and motion in random dot displays. *Vision Res* 1998; 38: 925–935. 9624441 10.1016/s0042-6989(97)00213-7

[33] Serrano-Pedraza I, Manjunath V, Osunkunle O, Clarke MPP & Read JCA. Visual suppression in intermittent exotropia during binocular alignment. *Invest Ophthalmol Vis Sci* 2011; 52: 2352–2364. 21220559 10.1167/iovs.10-6144

[34] Harris JM & Parker AJ. Efficiency of stereopsis in random-dot stereograms. *JOSA A* 1992; 9: 14–24. 10.1364/josaa.9.000014 1738046

[35] Kane D, Guan P & Banks MS. The limits of human stereopsis in space and time. *J Neurosci* 2014; 34: 1397–1408. 24453329 10.1523/JNEUROSCI.1652-13.2014 PMC3898296

[36] Guan P & Banks MS. Stereoscopic depth constancy. *Philos Trans R Soc B Biol Sci* 2016; 371: 20150253. 10.1098/rstb.2015.0253 PMC4901447 27269596

[37] Serrano-Pedraza I & Read JCA. Stereo vision requires an explicit encoding of vertical disparity. *J Vis* 2009; 9: 1–13. 19757912 10.1167/9.4.3

[38] Cumming BG & Parker AJ. Responses of primary visual cortical neurons to binocular disparity without depth perception. *Nature* 1997; 389: 280–283. 9305841 10.1038/38487

[39] Tittes J, Baldwin AS, Hess RF *et al*. Assessment of stereovision with digital testing in adults and children with normal and impaired binocularity. *Vision Res* 2019; 164: 69–82. 31377344 10.1016/j.visres.2019.07.006

[40] Adams WE, Leske DA, Hatt SR & Holmes JM. Defining real change in measures of stereoacuity. *Ophthalmology* 2009; 116: 281–285. 19091410 10.1016/j.ophtha.2008.09.012 PMC3903340

[41] Frisby JP, Mein J, Saye A & Stanworth A. Use of random-dot stereograms in the clinical assessment of strabismic patients. *Br J Ophthalmol* 1975; 59: 545–552. 1191612 10.1136/bjo.59.10.545 PMC1017407

[42] Fawcett SL & Birch EE. Validity of the Titmus and Randot circles tasks in children with known binocular vision disorders. *J AAPOS* 2003; 7: 333–338. 14566315 10.1016/s1091-8531(03)00170-8

[43] Vancleef K & Read JCA. Which stereotest do you use? a survey research study in the British Isles, the United States and Canada. *Br Ir Orthopt J* 2019; 15: 15–24. 32999970 10.22599/bioj.120 PMC7510382

[44] Read JCA, Rafiq S, Hugill J *et al*. Characterizing the Randot Preschool stereotest: testability, norms, reliability, specificity and sensitivity in children aged 2–11 years. *PLoS One* 2019; 14: e0224402. 31697704 10.1371/journal.pone.0224402 PMC6837395

